# Supplementary material for: Probing the Suitability of Different Ca2+ Parameters for Long Simulations of Diisopropyl Fluorophosphatase
Source: Molecules. 2021 Sep 26;26(19):5839. doi: 10.3390/molecules26195839 (PMC8510429; doi:10.3390/molecules26195839)
Supplement: Supplementary file 1 [file molecules-26-05839-s001.zip › molecules-1376275-supplementary.pdf]

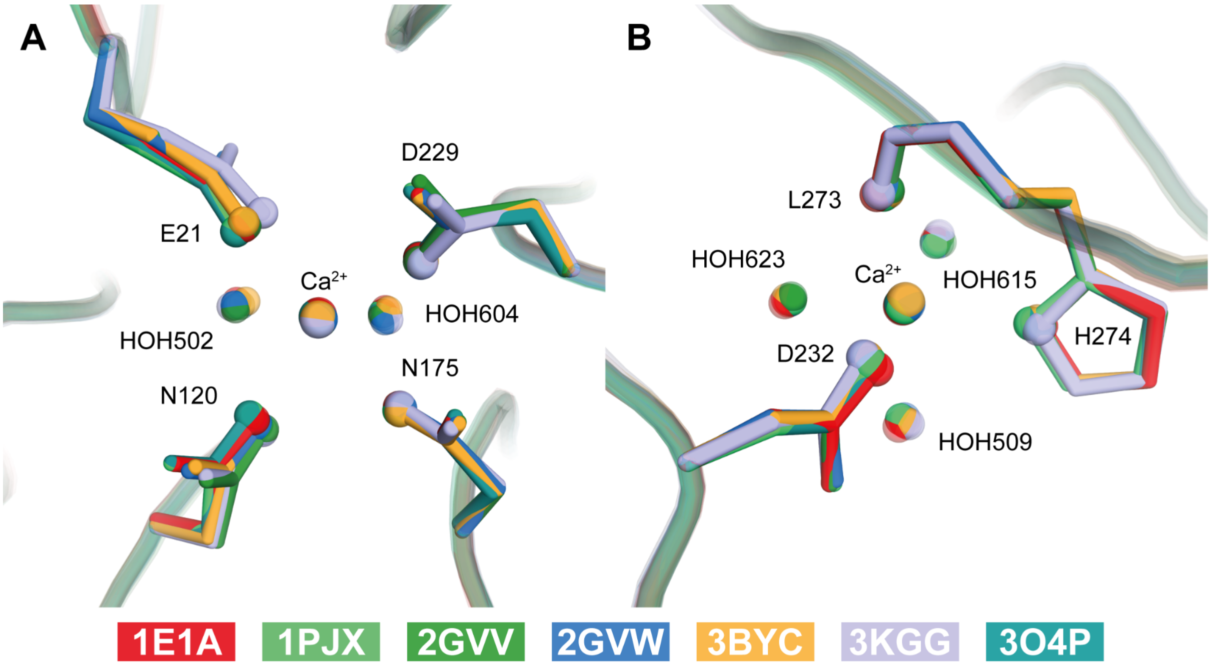

Figure S1. Low variability in the structure of calcium-binding sites of wild-type DFPase in X-ray structures. Water molecules are numbered according to 3O4P.

Table S1. Individual distance measurements demonstrate low variability in the conformation of the catalytic calcium binding site of wild-type DFPase in X-ray structures. Atoms used to calculate distances are shown as spheres in Figure S1. Distances are in angstroms.

PDB ID	E21	D229	N120	N175	HOH502	HOH604
1E1A	2.34	2.35	2.38	2.34	2.62	2.40
1PJX	2.35	2.36	2.33	2.40	2.48	2.35
2GVV	2.36	2.36	2.44	2.36	2.48	2.36
2GVW	2.37	2.32	2.42	2.34	2.49	2.33
3BYC	2.42	2.37	2.45	2.39	2.50	2.43
3KGG	2.35	2.42	2.49	2.30	2.64	2.44
3O4P	2.35	2.36	2.33	2.40	2.47	2.35
	2.36±0.02	2.36±0.03	2.41±0.06	2.36±0.03	2.53±0.07	2.38±0.04

Table S2. Individual distance measurements demonstrate low variability in the conformation of the structural calcium binding site of wild-type DFPase in X-ray structures. Atoms used to calculate distances are shown as spheres in Figure S1. Distances are in angstroms.

PDB ID	D232	L273	H274	HOH509	HOH615	HOH623
1E1A	2.22	2.34	2.56	2.41	2.28	2.35
1PJX	2.21	2.26	2.36	2.30	2.25	2.25
2GVV	2.20	2.34	2.52	2.36	2.32	2.23
2GVW	2.16	2.32	2.45	2.36	2.30	2.32
3BYC	2.16	2.23	2.43	2.42	2.43	2.38
3KGG	2.23	2.35	2.51	2.42	2.33	2.35
3O4P	2.21	2.26	2.37	2.30	2.25	2.25
	2.20±0.03	2.30±0.05	2.46±0.07	2.37±0.05	2.31±0.06	2.30±0.06

Table S3. Single-site nonbonded metal parameters, Gromacs notation. For all, the mass is 40.08 au and the charge is +2.

Model	σ, nm	ε, kJ/mol
**COM**	0.291320	0.40953
**IOD**	0.305497	0.36845
**HFE**	0.288116	0.19817
**Amber19 DEF**	0.305240	1.92376
**Charmm36m DEF**	0.243572	0.50208
**Oplsaa/m DEF**	0.241203	1.88136

Table S4. Charmm36m DEF NBFIX parameters for Ca2+ relevant for this study, Gromacs notation.

type	type	σ, nm	ε, kJ/mol	Comment
**CAL**	**CLA**	0.332037952251	0.561342176000	Calcium - Chlorine
**CAL**	**OC**	0.287938465703	0.502080000000	Calcium - ASP and GLU side chain oxygens

Table S5. Multisite dummy metal model parameters. Gromacs notation.

Nonbonded	**Mass, a.u.**	**Charge**	**σ, nm**	**ε, kJ/mol**
**dCa**	22.08	-1.0	0.2858	0.4323
**d1, d2, d3**	3.0	+0.5	0.0	0.0

Bonds	**func**	**b0, nm**	**kb, kJ/mol/nm^2**
dCa-d*	1	0.09	669440.0
d*-d*	1	0.1273	669440.0

Angles	**func**	**th0, deg**	**cth, kJ/mol/rad^2**
d1-dCa-d1	1	180.0	1046.0
d2-dCa-d2	1	180.0	1046.0
d3-dCa-d3	1	180.0	1046.0
d1-dCa-d2	1	90.0	1046.0
d1-dCa-d3	1	90.0	1046.0
d2-dCa-d3	1	90.0	1046.0
dCa-d1-d2	1	45.0	1046.0
dCa-d1-d3	1	45.0	1046.0
dCa-d2-d1	1	45.0	1046.0
dCa-d2-d3	1	45.0	1046.0
dCa-d3-d1	1	45.0	1046.0
dCa-d3-d2	1	45.0	1046.0
d1-d2-d1	1	90.0	1046.0
d1-d3-d1	1	90.0	1046.0
d2-d1-d2	1	90.0	1046.0
d2-d3-d2	1	90.0	1046.0
d3-d1-d3	1	90.0	1046.0
d3-d2-d3	1	90.0	1046.0
d1-d2-d3	1	60.0	1046.0
d1-d3-d2	1	60.0	1046.0
d2-d1-d3	1	60.0	1046.0

Dihedrals	func	phase, deg	kd, kJ/mol/rad^2	pn
d*-X-X-X	9	0	0	1
X-d*-X-X	9	0	0	1
dCa-X-X-X	9	0	0	1
X-dCa-X-X	9	0	0	1

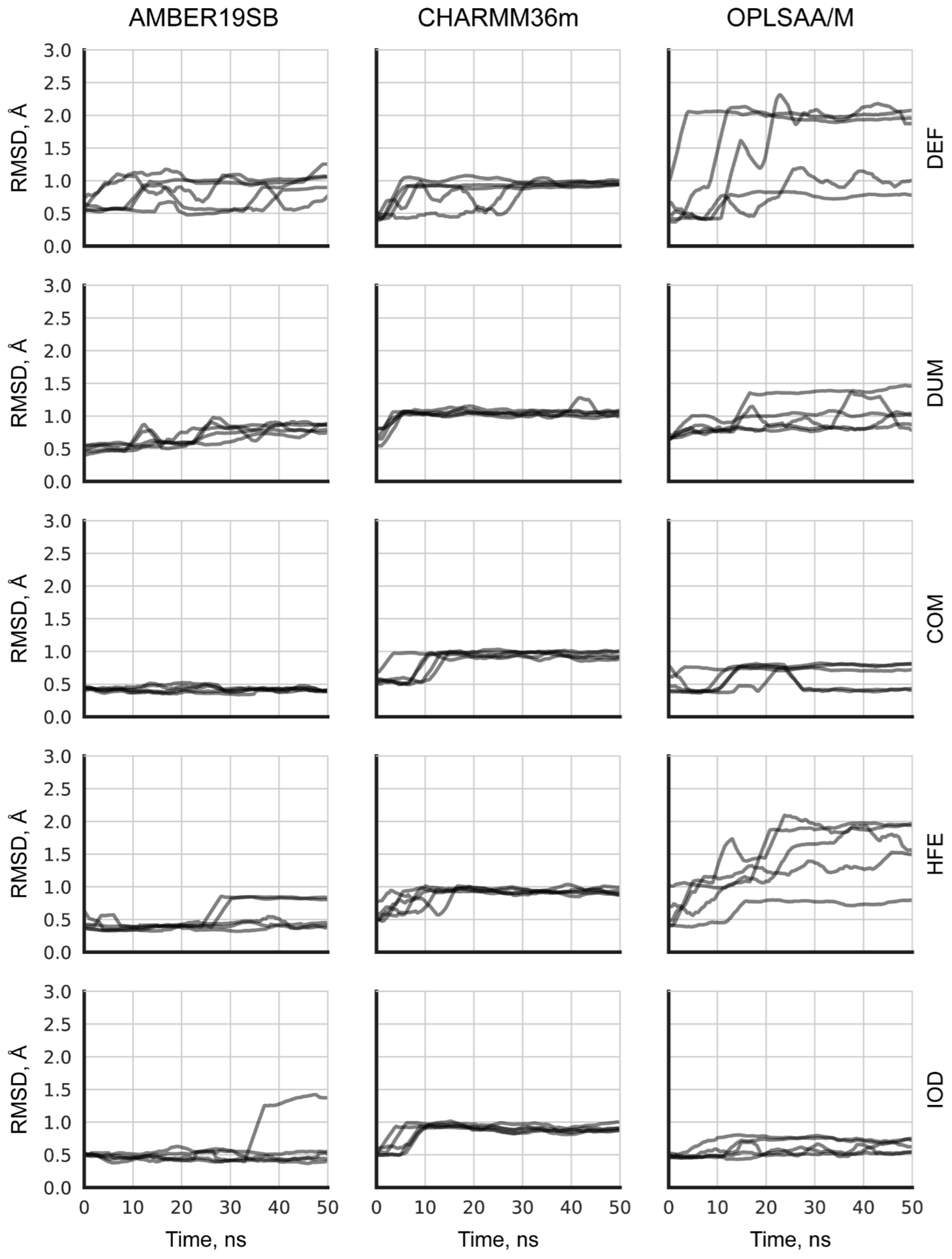

Figure S2. Time evolution of catalytic Ca^{2+} site displacements in individual simulations.

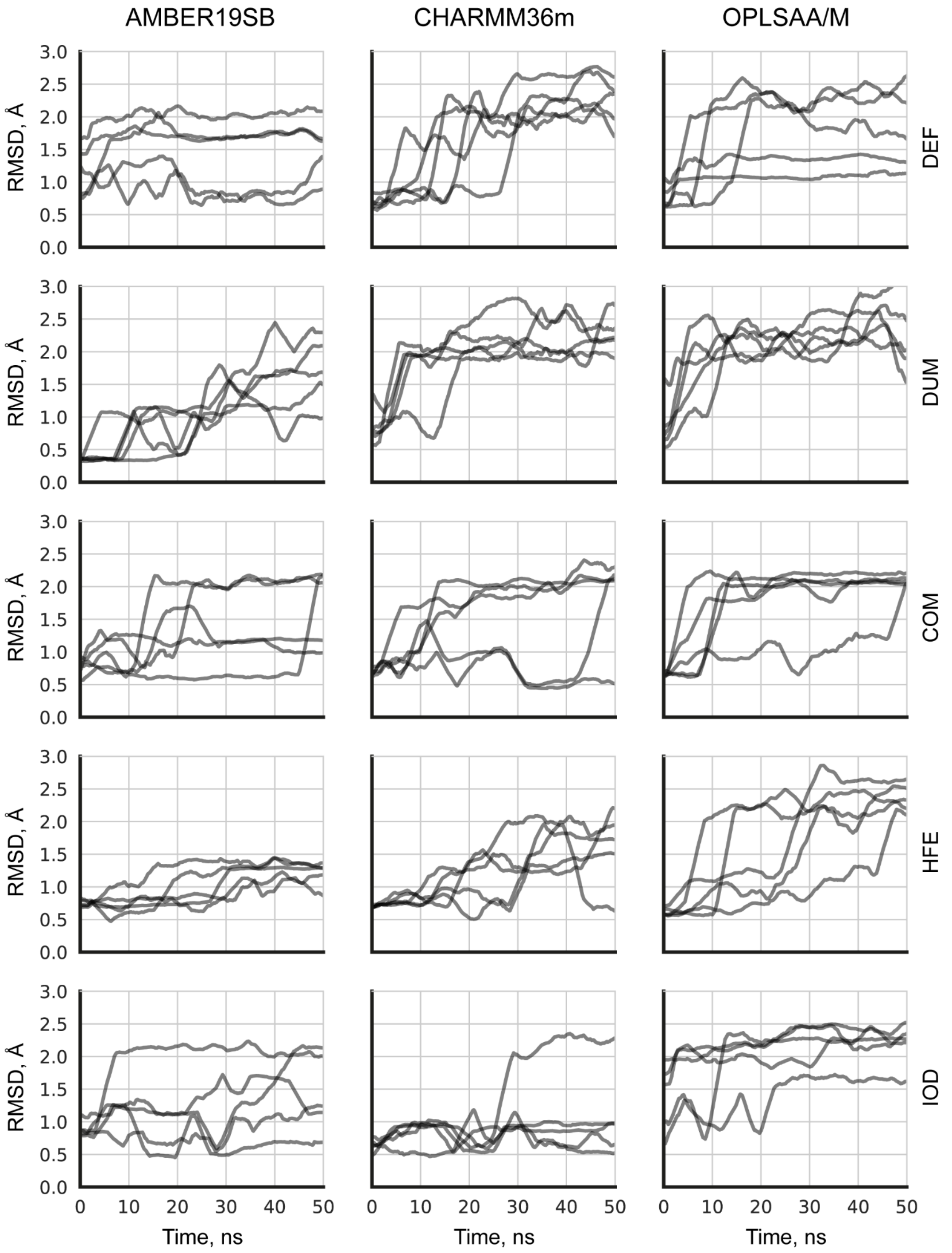

Figure S3. Time evolution of structural Ca^{2+} site displacements in individual simulations.

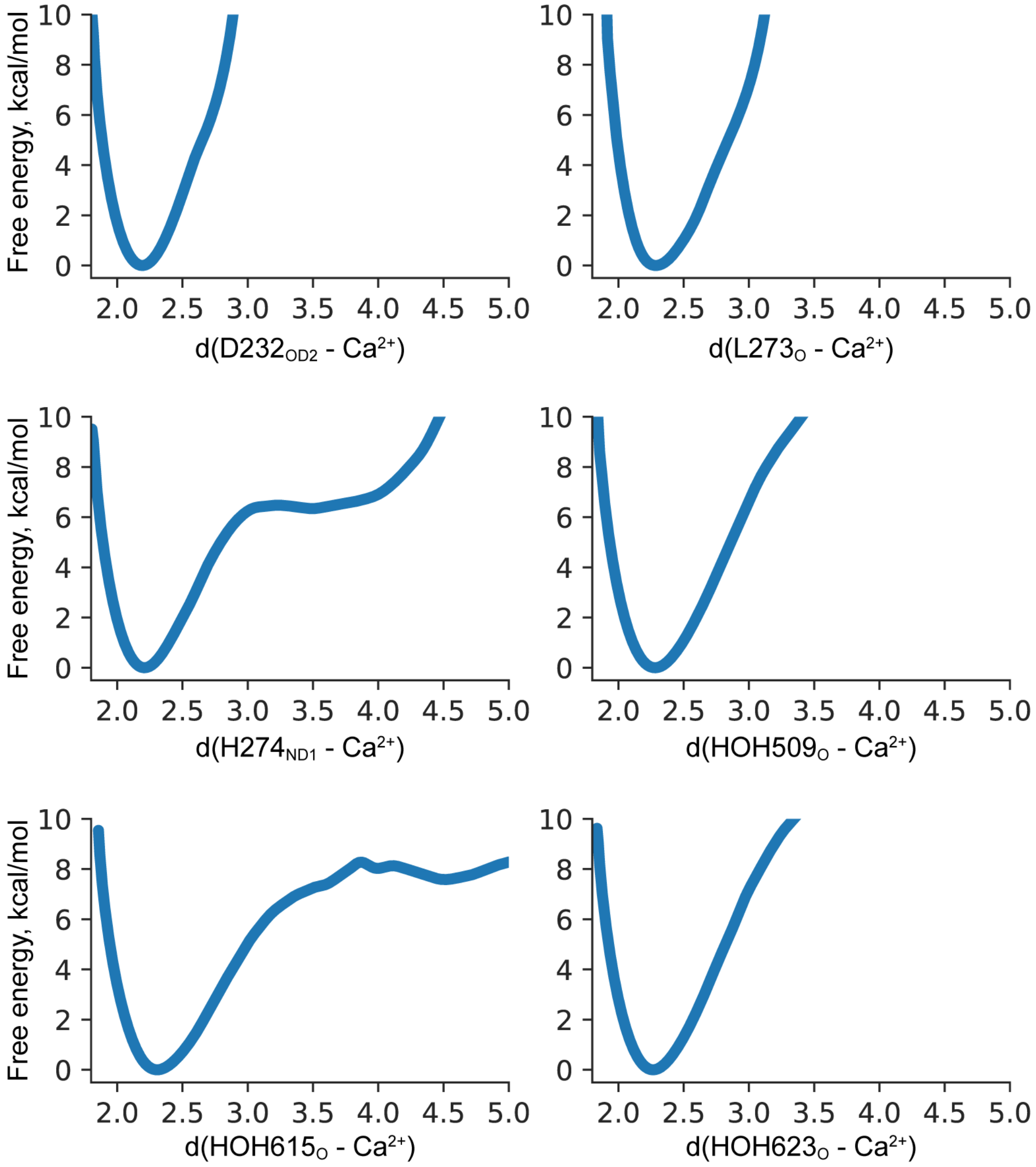

Figure S4. Free energy profiles of interactions between structural Ca^{2+} and its coordinators. Water molecule numbering corresponds to PDB ID 3O4P.

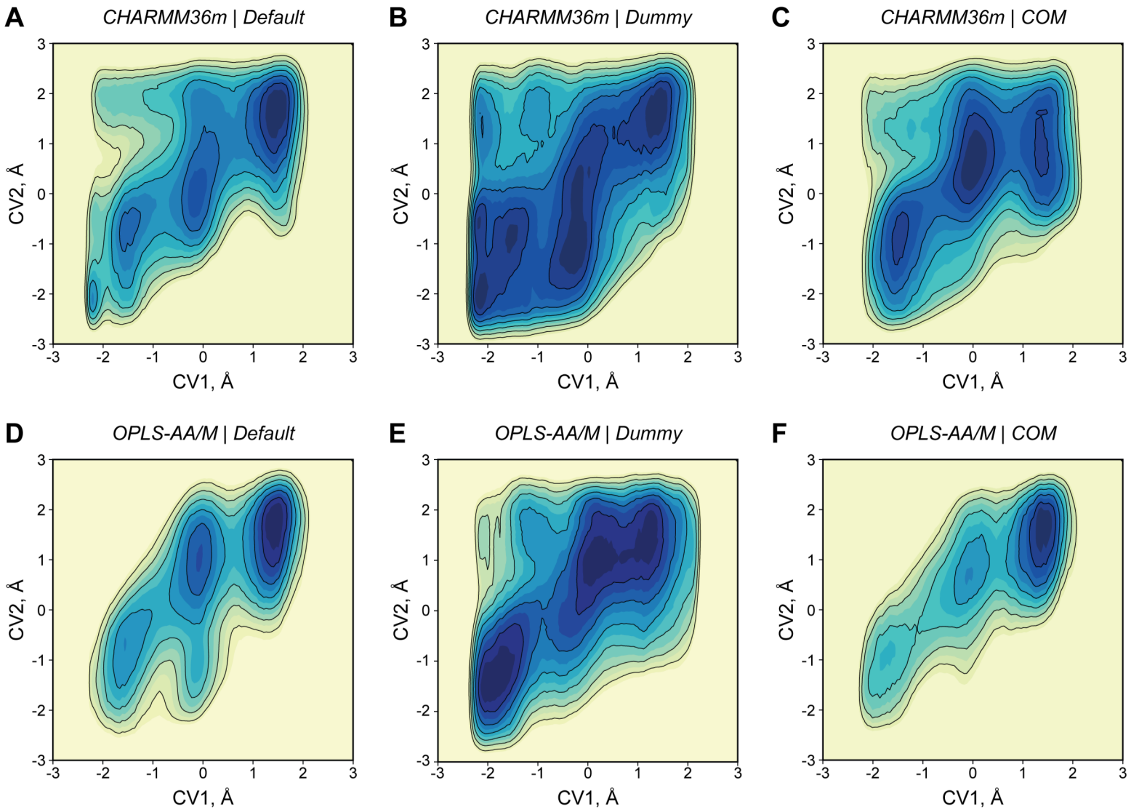

Figure S5. Competition for hydrogen bond with G22 and its influence on E21 conformation in CHARMM36m and OPLS-AA/M simulations. A-C. CHARMM36m parameter combinations. D-F. OPLS-AA/M parameter combinations.

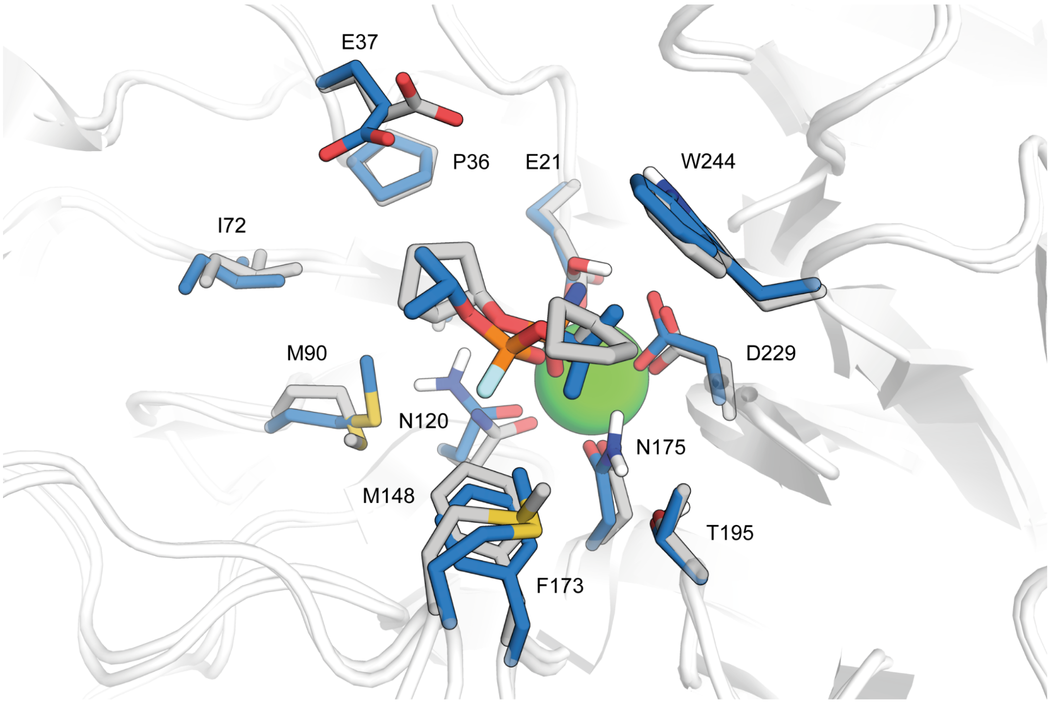

Figure S6. Comparison of ligand binding poses from our study with DFP (blue) and inhibited complex 2GVV (gray).
